# Genetic Drivers of Pancreatic Cancer Are Identical Between the Primary Tumor and a Secondary Lesion in a Long-Term (>5 Years) Survivor After a Whipple Procedure

**DOI:** 10.1089/pancan.2018.0015

**Published:** 2018-11-01

**Authors:** Tyler M. Bauer, Teena Dhir, Adam Strickland, Henry Thomsett, Austin B. Goetz, Shawnna Cannaday, Jonathan R. Brody, Michael J. Pishvaian, Charles J. Yeo

**Affiliations:** ^1^ Sidney Kimmel Medical College, Thomas Jefferson University, Philadelphia, Pennsylvania.; ^2^ Department of Surgery, The Jefferson Pancreas, Biliary, and Related Cancer Center, Thomas Jefferson University, Philadelphia, Pennsylvania.; ^3^ Lombardi Comprehensive Cancer Center, Georgetown University, Washington, District of Columbia.

**Keywords:** KRAS, metachronous, MYC, pancreatic ductal adenocarcinoma, pancreaticoduodenectomy, Whipple procedure

## Abstract

**Background:** A new mass in the remnant pancreas of a patient with previously resected pancreatic ductal adenocarcinoma (PDA) typically represents either a recurrence of the initial primary tumor or a second primary tumor. Recent advances in next-generation sequencing (NGS) strategies allow us to compare the genetic makeup of primary and secondary lesions.

**Case presentation:** A 50-year-old Caucasian female presented for a surgical evaluation of a new biopsy-proven PDA at the junction of the body and tail of the pancreas. Six years prior, in 2011, the patient was found to have a T3N0M0 PDA of the pancreatic head, which was surgically resected with a classic Whipple procedure and concurrent hemicolectomy. Pathology showed pancreatic intraepithelial neoplasia grade 2 and PDA with negative surgical margins, positive perineural spread, and negative lymphovascular spread, and the patient received adjuvant chemotherapy and local radiation. In 2017, she was diagnosed with a new PDA lesion in the remaining pancreatic body far from the previous anastomosis site and was taken to surgery for a completion pancreatectomy and revision of the gastrojejunostomy. NGS was performed on both specimens. Both lesions shared identical mutations in *KRAS*, *TP53*, and *CDKN2A* genes. Amplifications of *MYC* and mutant *KRAS* were identified in the 2017 tumor and an *ACVR1B* mutation was identified in the 2011 tumor, but was not found in the 2017 tumor.

**Conclusions:** This case demonstrates the ability to evaluate similarities between key genetic drivers from a resected primary tumor and a PDA lesion that presented in the same patient 6 years later. Histological analysis and NGS can be used to understand potential differences and similarities between lesions and may be useful in future studies as predictive markers or to provide insight into resistance mechanisms (e.g., MYC amplification).

## Introduction

Pancreatic ductal adenocarcinoma (PDA) accounts for 45,000 cancer diagnoses in the United States per year and 90–95% of all pancreatic cancers.^1–3^ PDA has historically high rates of recurrence and the lowest 5-year survival of any cancer.^2^ Only 20% of patients are eligible for resection upon initial presentation,^4^ with an average postresection survival of 16–24 months; 5-year survival postresection is ∼20–35%.^5,6^ Surgical candidates classically received adjuvant gemcitabine, although emerging data involving the use of adjuvant mFOLFIRINOX may be superior, with a median overall survival of ∼54 months.^7^ Predictors of poor survival include positive surgical margins, positive lymph node metastasis, large tumor size (>3 cm), and poor histological differentiation.^5,8^ An unfortunate reality of treating pancreatic cancer is that even after 5 years of disease-free survival, recurrent disease is still possible, with ∼20% of patients who survive 5 years having recurrences (both locoregional and distant).^6^ Resection of locally recurrent lesions is safe and effective in selected individuals.^9,10^

To fully understand this disease, it is important to understand PDA's biology, including key driver mutations leading to the progression of normal pancreatic tissue to PDA. These driver mutations include mutations in *KRAS* and *TP53* genes, with over 95% of PDA cases having a *KRAS* mutation (G12D most common).^11^ There is a clear link between *KRAS* mutations and inactivation of important tumor suppressors, such as *CDKN2A* and *TP53*, which causes the progression of pancreatic intraepithelial neoplasia (PanIN) lesions to PDA; 90% of low-grade PanIN-1A lesions have a *KRAS* mutation, which is evidence that it is a driver mutation at an early stage in PDA.^12^ After progression to PanIN-1B, 95% of lesions acquire a mutation in *CDKN2A*, and upon progression to PanIN-3, 75% of tumors acquire a *TP53* mutation, and 55% attain an *SMAD4* mutation.^11,13^ Identifying these mutations and patients with high risk of developing these mutations is important as it is these patients who are at higher risk of developing cancer and recurrence.

## Case Presentation

### Clinical background

A 50-year-old Caucasian female, with no previous smoking history or pancreatic cancer family history, presented to our clinic for surgical evaluation of a biopsy-proven PDA noted at the junction of the body and tail of the pancreas. The patient had previously undergone a classic pancreaticoduodenectomy (Whipple) procedure with concurrent hemicolectomy 6 years prior (in 2011) for a T3N0M0 adenocarcinoma of the pancreatic head that had invaded the mesentery of the proximal transverse colon. After the 2011 surgery, pathology revealed a poorly differentiated PDA along with a PanIN grade 2. The final pathology showed negative surgical margins, positive perineural spread, and 0/33 specimen lymph node involvement. From June 2012 to October 2012, the patient underwent and completed adjuvant chemotherapy with gemcitabine, capecitabine, and radiation at an outside hospital. She was carefully followed by her medical oncology team with serial CA 19-9 monitoring and abdominal MRIs on an ongoing basis. Due to her young age, in 2011, she underwent genetic screening and no germline mutations were identified. Since the time of the primary resection, the patient had been high functioning and healthy, with the exception of some problems of early satiety and recurrent cholangitis. These sequelae were attributed to close proximity of the gastrojejunostomy and hepaticojejunostomy, with possible reflux of intestinal contents up the afferent limb, all partially managed by diet changes.

**FIG. 1.**
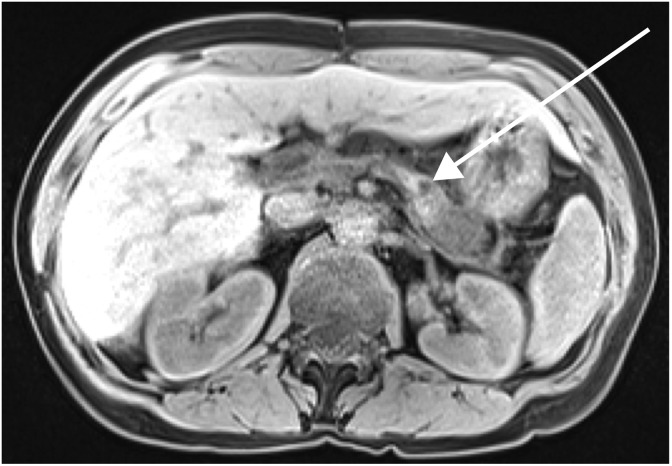
MRI (axial T1 weighted) of the 2017 pancreatic mass. The image was read to show interval development of a mass within the pancreatic body–tail junction with restricted diffusion and hypoenhancement. The mass was measured to be 2.3 × 2.2 cm (see arrow), and it was felt to focally encase the splenic artery and segmentally narrow the splenic vein.

**FIG. 2.**
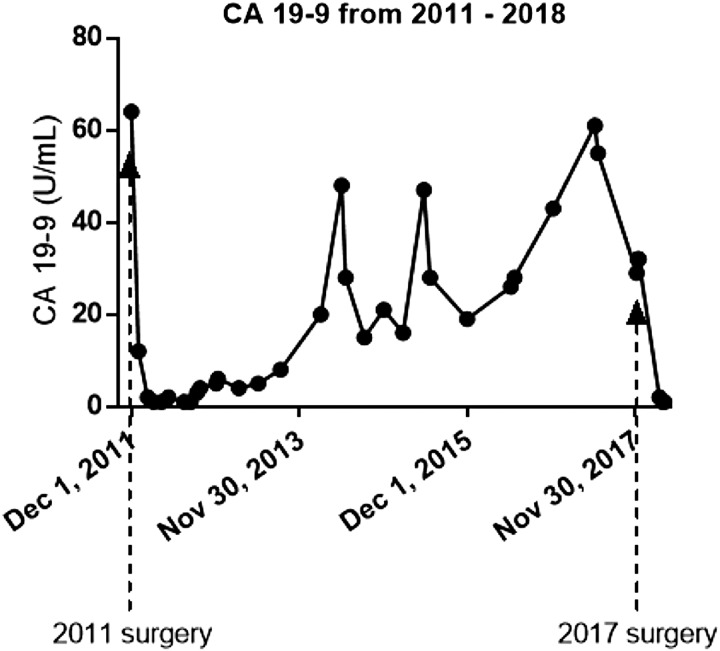
Chronologic results of CA 19-9. Results are listed starting before the initial tumor resection in 2011 to identification of the new tumor in the pancreatic remnant in 2017.

In 2017, a biannual screening MRI with intravenous contrast showed a new pancreatic lesion measuring 2.3 × 2.2 cm in the tail of the pancreas (Fig. 1). Esophagogastroduodenoscopy and endoscopic ultrasound-guided biopsy identified it as a poorly differentiated adenocarcinoma. From 2011 to 2017, she had had close followup with serial CA 19-9, and a measurement of this marker after identification of the lesion on MRI showed an elevation, which was confirmed on repeat testing (61 and 55 U/mL; normal <37 U/mL). This was the first instance of two consecutive CA 19-9 measurements outside of the normal range since resection of the primary cancer 6 years prior. When the patient presented to our institution a few months later, the CA 19-9 had returned to normal at 32 U/mL (Fig. 2), and there was a moderate increase in CEA (16.9 ng/mL; normal <3 ng/mL). The patient received a second MRI of the abdomen and pelvis with contrast to identify distant disease, which showed the lesion to be confined to the pancreas. Along with the MRI of the abdomen, a CT of the chest was preformed, which showed no gross metastatic lesions. A completion pancreatectomy was scheduled with revision and lengthening of the jejunal limb proximal to the gastrojejunostomy to resect the tumor and treat her episodes of early satiety and recurrent cholangitis.

### Surgical intervention

**FIG. 3.**
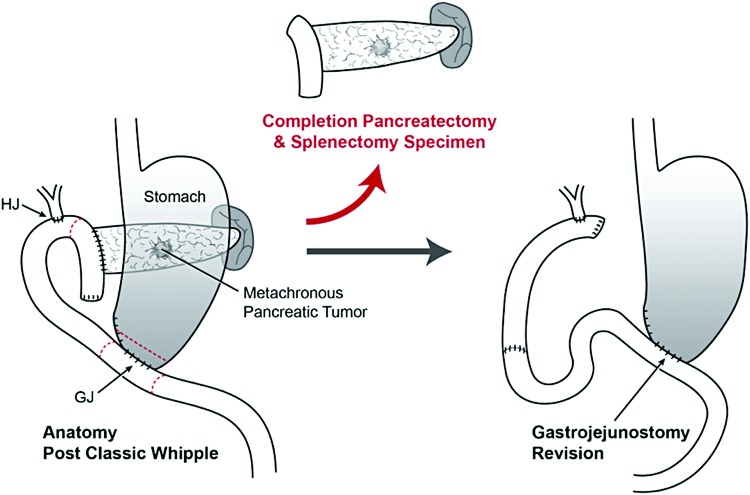
Operative procedure summary; left depicting the patient's postclassic Whipple procedure anatomy (2011 surgery) and right depicting the patient's postrevision completion pancreatectomy anatomy (2017 surgery). Gastrojejunostomy was revised to address the patient's early satiety and cholangitis, which was thought to be due to a short afferent limb.

Intraoperatively, the patient's three Whipple anastomoses were noted to be grossly intact and there was no evidence of metastatic disease. First, the stomach was divided approximately two centimeters proximal to the prior gastrojejunostomy. The jejunostomy was closed, and gastrocolic and gastrosplenic ligaments were divided. The splenic artery was ligated, and the splenocolic ligament was divided. The spleen and pancreas were mobilized out of the retroperitoneum. The jejunum was divided between the pancreaticojejunostomy (PJ) and hepaticojejunostomy. The proximal jejunum, prior PJ, remaining pancreas, and spleen were removed. The tumor was noted to be grossly confined to the pancreas. The distal end of the stomach was delivered through the mesocolon and a retrocolic gastrojejunostomy was undertaken 60 cm downstream from the hepaticojejunostomy. The anatomy before and after this operation is shown in Figure 3.

### Postoperative course

The patient tolerated the procedure well and had an uncomplicated hospital course. The patient was closely followed postoperatively and did well. She completed two cycles of adjuvant chemotherapy with gemcitabine and capecitabine from January 2018 to April 2018, but did have some neutropenia at the end of her adjuvant therapy requiring pegfilgrastim. Her intermittent fevers, early satiety, and abdominal pain resolved after the surgery. She became an obligate insulin-dependent diabetic after the completion pancreatectomy procedure and now requires exogenous pancreatic enzymes to support her nutrient absorption.

### Pathology and molecular analysis

The specimen was found to be consistent with a poorly differentiated invasive adenocarcinoma. Resection margins were negative, and 2 of 17 lymph nodes were positive for metastatic cancer. We sent representative slides of the patient's 2017 tumor and 2011 tumor to Perthera (McLean, VA) for next-generation sequencing (NGS) and histological analysis, which tested for mutations in a total of 315 genes and stained for various predictive biomarkers (Table 1). Both lesions showed the same mutations in *KRAS* (G12R), *CDKN2A* (splice site 151-1 G to A), and *TP53* (Y220C). In addition, her 2011 tumor had a mutation in *ACVR1B* (S4) that was not present in the 2017 tumor, and the 2017 tumor had amplifications of *MYC* and mutant *KRAS* that were not present in the 2011 tumor (Table 1). Upon histological analysis, staining for MLH1, MSH2, MSH6, PMS2, pAKT, and HER2 was similar in both samples, but in the 2017 tumor, there was 60% increased staining for RRM1 and 20% increased staining for ERCC1, which changed the classification from low to high staining for ERCC1.

**Table 1.** **Summary of Next-Generation Sequencing on Both 2011 and 2017 Tumor Samples**

2011 Sample			2017 Sample			
Genes tested	Mutation	Mutation allele frequency (%)	Gene tested	Mutation/amplification	Mutation allele frequency (%)	Amplification copy number
KRAS	G12R	15.3	KRAS	G12R	88.3	38
ACVR1B	S4	14.9	ACVR1B	(No mutation identified, wild type)		
CDKN2A	Splice Site 151-1G > A	14.4	CDKN2A	Splice site 151-1G > A	52	
TP53	Y220C	10.3	TP53	Y220C	44	
MYC	(No mutation identified, wild type)		MYC	Amplification		76

## Discussion

A patient who has had surgical resection of pancreatic adenocarcinoma is at a high risk for recurrence, even with a seemingly adequate surgical intervention; more than 70% of patients with negative margins experience recurrence.^6^ The majority of these recurrences happen early, although there are reports of recurrence even after 5 years of disease-free survival.^6^ Some surgical specimen characteristics have been shown to influence mortality, such as positive surgical margins, positive lymph node metastasis, large tumor size (>3 cm), and poor histological differentiation^5,8^; however, in resections that achieve R0 status, it seems that the presence of synchronously adjacent PanIN lesions in the surgical specimen (regardless of grade) does not influence outcomes.^14,15^ There are no clear guidelines on how to screen for a recurrent PDA in postoperative pancreatic cancer patients.^16^ It is imperative to closely monitor these patients for signs of local and distant recurrence in the surgical bed as well as the liver, lungs, and peritoneal cavity, which are the most common sites of metastasis; this is most commonly achieved with CT.^17^ Our patient had a new lesion that was identified on MRI monitoring in 2017. Since our patient had a followup MRI of the abdomen and pelvis and CT of the chest that showed no metastatic disease, a completion pancreatectomy was planned, although a PET scan could have been utilized to rule out metastatic disease as well. For patients with no evidence of distant metastatic disease and an apparent pancreas-confined lesion, as was the case with our patient, resection of the new lesion can be safe and effective.^9,10^

It is often unclear if a new lesion that occurs in an organ after extended cancer-free survival represents a recurrent lesion or a second primary/metachronous cancer. The concept of a field defect was first introduced in 1957 by Slaughter et al. and describes an underlying genetic defect of an organ or developmental field that predisposes that tissue to cancer.^18^ Subsequently, it has been shown that field defects can cause shared genetic mutations of large patches of tissue and, in extreme cases, entire organs.^19^ While initially described in oral epithelial cancers, the concept of field defects has been reproduced in many different organ systems, including the pancreas.^20^ Field defects have been implicated in cases of second primary tumors (also referred to as second field tumors).^20^ Second primary tumors tend to occur at sites distant to the primary resected surgical site, as opposed to recurrences that are adjacent to the surgical scar.^20^ When assessing whether a lesion is a second primary or recurrent lesion, it is important to remember that genetically the recurrent tumor should be near identical to the original. In the case of a second primary tumor, there can be new mutations that are unique to the second primary tumor, and mutations present in the primary tumor may not be represented in the second tumor.^19,20^

Due to identification of the patient's new lesion many years following the initial procedure, which had negative margins, and the fact that the mass was not noted at the margins of the prior procedure, it was our clinical suspicion that this may be a case of a metachronous cancer. Our genetic testing confirmed that the new tumor in 2017 had similar genetic mutations in *KRAS*, *TP53*, and *CDKN2A* compared with the 2011 cancer, but the mutation in *ACVR1B* was not present in the 2017 sample, and mutant *KRAS* (copy number 38) and *MYC* (copy number 76) amplifications were only present in the 2017 sample. Without further and more sophisticated genetic testing, we cannot determine precisely if the new tumor represents a second primary tumor or a recurrent tumor.

*KRAS* and *MYC* have been extensively studied with regard to cancer. *KRAS* mutations are present in over 90% of pancreatic cancers and are noted as being the earliest genetic alterations in majority of these cancers.^12^ *KRAS* encodes a proto-oncogene GTPase, which (when mutated) results in constitutively activated signaling pathways leading to sustained proliferation and antiapoptosis.^21^ MYC is a transcription factor and is implicated in over 450,000 American patients with cancers per year.^22^ MYC acts as a sensor, integrating cellular signals and ultimately affecting cellular responses, including differentiation, survival, and pluripotency.^23^ MYC has a short half-life in the cell, estimated to be 20–30 min, and its production and degradation are tightly regulated by transcription factors, ubiquitin proteasome degradation, and micro-RNA.^24–26^ Mutations that result in amplification or overexpression of *MYC* result in tumorigenesis.^23,27^

*MYC* and *c-MYC* deregulation is common in PDA, with ∼30% of primary and metastatic tumors showing *MYC* amplification.^28^ In a small sample size, Schleger et al. were able to identify two patients with high *MYC* amplification in the metastatic site when compared with the primary tumor, which had low *MYC* amplification.^28^ There was strong correlation between *MYC* amplification and protein expression and tumor grade in this population as well, demonstrating its role in PDA progression.

Although there is limited literature published in regard to *MYC* and *KRAS* amplifications in pancreatic metachronous tumors, in breast cancer, *c-MYC* amplification is associated with tumor progression, earlier tumor relapse, and worse overall survival.^29^ In patients with recurrent breast cancer tumors with *c-MYC* amplification, it has been found that half of these patients with breast cancer lose their hormone receptor expression and overall have shorter recurrence-free survival when compared with patients with normal *c-MYC* status.^29^ It was also found that *MYC* amplification is a risk factor for developing chemoresistance in patients with breast cancer, except for chemotherapeutics such as doxorubicin and cyclophosphamide.^29^ In endometrial cancer, *KRAS* amplification has been seen in 18% of metastatic lesions compared with only 3% in primary tumors (*p* < 0.001), and amplification status correlates with poor outcome, higher grade, and lost hormone receptor expression.^30^

In our patient, it is possible that the patterns of mutations and amplifications seen in the 2011 and 2017 tumors are due to a field defect or genetic vulnerability involving the entire pancreas. NGS analysis of the two tumors showed an amplification of *MYC* with a copy number of 76 in the 2017 tumor, which was not present in the 2011 tumor, along with a significant mutant *KRAS* amplification. Furthermore, it stands to reason that the shared *KRAS*, *TP53*, and *CDKN2A* mutations were the direct result of a field defect of the pancreas, while *MYC* amplification was not.

Another possibility that explains these genetic similarities and differences is the characteristic of tumor heterogeneity. Three identical driver mutations were present in both of the patient's lesions, which may be indicative of a recurrence/metastasis. In this case, loss of the *ACVR1B* mutation can be reconcilable by tumor heterogeneity, or a later selected mutation, or simply a passenger mutation. PDA, in general, has a high degree of genetic heterogeneity, with varying degrees of mutations from one area of the tumor to the other, which can also influence chemotherapy response.^31,32^ Over the time course of tumor progression, separate cancerous foci can progress to PDA (i.e., clonal progression), but due to varying degrees of genetic heterogeneity, each clone can have a different genetic makeup even though they all originated from the same original clone.^31^ Thus, it is possible that due to selection and sampling bias during NGS, the *MYC* and *KRAS* amplifications were not identified, but were present in both the 2011 and 2017 samples. In addition, these amplifications can occur purely due to the 6-year time period between tumor developments and may be an example of normal PDA progression over time. In an article by Yachida et al., genetic analysis of a patient's primary and metastatic tumors showed an average of 64% of mutations to be due to founder mutations (present in the original clone) and an average of 36% of mutations to be due to progressor mutations (not present in the original clone) with clonal evolution and metastasis.^32^ These progressor mutations, such as *MYC* and *KRAS* amplification in our case, were not found in the parental clone and can be considered to either be passenger or driver mutations and could have possibly contributed to development of the 2017 tumor. Many hypotheses can be made in regard to the reason why the NGS is different between the 2011 and 2017 samples, so their results should be taken in context. One could seek to elucidate the true nature of the patient's second lesion with additional tests to demonstrate clonality, such as single-nucleotide polymorphism arrays or X-gene imprinting, although this was not feasible in our case due to limited amount of viable specimen present.

Although the 2011 sample had negative resection margins and lymph node status, microdissemination could have occurred and led to the 2017 tumor development, favoring the argument that the 2017 tumor is a result of recurrence and not a metachronous tumor. As highlighted in this case, the use of NGS is a tool and should be used as an adjunct in diagnosis, but it does not serve as a stand-alone test to distinguish between recurrence and metachronous lesions. This case report brings to light the importance of clinical correlation when interpreting genetic testing results.

Although the data gathered from genetic testing are invaluable, we would like to discuss some logistical challenges that we encountered during this process. First, when it was identified that this patient was rediagnosed with PDA in 2017, it was difficult to obtain the 2011 tumor sample for comparison as over the past 6 years the patient had received care at multiple different hospitals. Second, the 2011 tumor slides had a heavy necrotic burden, so many of them could not be used for NGS, and fragments of the tumor from multiple slides were needed to allow for enough usable DNA. Third, lack of coordination between different institutions' medical records, pathology, and insurance approval for testing also led to delays in obtaining sequencing results. In the end, the samples were able to be processed, but it is important to remember that without assistance from members in all these departments and institutions, these genetic findings would be not have been successfully identified.

## Conclusion

We present a case of a 50-year-old female who has experienced two instances of PDA at a young age. With the use of NGS, we were able to perform genomic profiling of both lesions, which identified both retained and unique mutations between the two samples. With the limited NGS data we obtained, we cannot definitively conclude the molecular etiology of this secondary tumor. Although the distinction between recurrent and metachronous lesions can be vague, we can confidently write that common genetic drivers between the 2011 and 2017 tumors are completely identical. Therefore, in a hypothetical case where these genetic drivers are actionable, they could have clinical value in the future as potential predictive and/or resistance markers for next-line therapy.

## Abbreviations Used

NGS: next-generation sequencing
PanIN: pancreatic intraepithelial neoplasia
PDA: pancreatic ductal adenocarcinoma
PJ: pancreaticojejunostomy
